# A new method for quantification of aortic stiffness in vivo using magnetic resonance elastography (MRE): a translational study from sequence design to implementation in patients

**DOI:** 10.1186/1532-429X-17-S1-O42

**Published:** 2015-02-03

**Authors:** Rachel Clough, Ondrej Holub, Henry Fok, Nicholas Gaddum, Jordi Alastruey, Ralph Sinkus

**Affiliations:** 1 King's College London, London, UK

## Background

Aortic stiffness is an important risk factor in the development of cardiovascular disease. Early detection of stiffening is important so that appropriate medical and behavioural interventions can be implemented. Applanation tonometry pulse wave velocity is the current non-invasive reference standard but this only provides spatially-averaged measurements. Local measurements may be more important, particularly in the ascending aorta due to aorto-ventricular coupling effects. MRE usually requires a surface transducer to generate shear waves in the tissues but these are expensive, uncomfortable and not available in all centres.

The aim of this study was to develop a new transducer-free MRE sequence to measure aortic stiffness *in vivo* using aortic valve closure, an intrinsic source for elastography, to generate shear waves in the aortic wall.

## Methods

This study has 4 parts: sequence development (pulse programming and simulations) and implementation on a clinical 3T MR scanner; validation of MRE measurements in (a)simple geometry and (b)aortic phantoms; a volunteer study (n=8) to assess cardiac and respiratory motion compensation strategies and the sensitivity of the motion-encoding gradients; and a patient study (hypertensive patients, n=15) to compare MRE and other MR methods (QA Loop; transit-time (TT)) for measurement of aortic stiffness to the current non-invasive reference standard applanation tonometry.

## Results

The MRE sequence was successfully developed and implemented. Phantom experiments demonstrated MRE could measure the true stiffness of the material and wave-guidance effects (a:4.6m/s; b:4.0m/s). A 1D respiratory navigator with ECG-gating provided satisfactory motion compensation in all volunteers. Motion-encoded images (165Hz) demonstrated aortic wall shear waves associated with valve closure at 375ms (277-420) after the R-wave. MRE successfully provided local measurements of aortic stiffness in patients and in contrast to the other methods, could specifically assess the ascending aorta. MRE showed the greatest correlation and agreement with the reference standard (R^2^=0.78(95%CI 0.69-0.96) p<0.0001; bias-1.3m/s(95%LoA -4.43-1.79)) compared with other MR Methods

TT- tonometry (R^2^=0.40(0.009-0.90); p=0.048; bias-1.6m/s(-5.30-2.01)) and QA Loop- tonometry (R^2^=0.002(-0.46-0.52) p=0.87; bias-3.8m/s(-8.94-1.31)).

## Conclusions

We have successfully developed, validated and applied a new method to quantify aortic stiffness *in vivo*. We have shown for the first time that shear waves are generated in the aortic wall by aortic valve closure, and these can be measured using our novel MRE technique. This methodology, which includes local measurements in the ascending aorta, can be easily translated to other centres because a transducer is not required. MRE has the potential to become an important screening test for early detection of cardiovascular disease and to risk stratify and optimise treatment for individual patients.

## Funding

BHF/Wellcome Trust/MRC/AMS.

